# A model of evolution and structure for multiple sequence alignment

**DOI:** 10.1098/rstb.2008.0170

**Published:** 2008-10-07

**Authors:** Ari Löytynoja, Nick Goldman

**Affiliations:** EMBL-European Bioinformatics InstituteHinxton, UK

**Keywords:** sequence alignment, insertion–deletion processes, character homology, evolutionary process heterogeneity

## Abstract

We have developed a phylogeny-aware progressive alignment method that recognizes insertions and deletions as distinct evolutionary events and thus avoids systematic errors created by traditional alignment methods. We now extend this method to simultaneously model regional heterogeneity and evolution. This novel method can be flexibly adapted to alignment of nucleotide or amino acid sequences evolving under processes that vary over genomic regions and, being fully probabilistic, provides an estimate of regional heterogeneity of the evolutionary process along the alignment and a measure of local reliability of the solution. Furthermore, the evolutionary modelling of substitution process permits adjusting the sensitivity and specificity of the alignment and, if high specificity is aimed at, leaving sequences unaligned when their divergence is beyond a meaningful detection of homology.

## 1. Introduction

Sequence alignment aims to match homologous characters, nucleotides or amino acids that are descended from a common ancestor. This is complicated by base substitutions that decrease similarity between sequences over evolutionary time and insertions and deletions that add and remove sequence in different evolutionary lineages. From the end user's point of view, the sequence alignment problem is about placing homologous residues in the same alignment columns and positioning gaps to indicate inserted and deleted sequence.

Depending on the aim of the analysis, sequences in an alignment can be seen as descendants of an ancestral sequence or a set of sequences sharing a common or a related biological function. Hence, multiple sequence alignment methods have traditionally modelled either the hierarchical relationships among the sequences (Hogeweg & Hesper 1984; Thompson *et al*. 1994) or the varying structural and functional constraints along the sequence sites (Eddy 1998; Karplus *et al*. 1998). There have been few attempts to combine the two alternative approaches (e.g. Edgar & Sjölander 2003; Arribas-Gil *et al*. 2007), but so far these methods have been either not suitable for alignment of several sequences and genome-scale analyses or computationally too hard to be biologically realistic (e.g. Satija *et al*. 2008).

We present a method that combines the strengths of tree- and profile-based alignment algorithms and simultaneously describes the evolution and regional heterogeneity, from here on called *sequence structure*, of multiple sequences. Our approach is based on a pairwise alignment model that consists of a moderate number of evolutionary processes, each describing a set of differently evolving sequence sites or a sequence region. Distinct processes are depicted with structure classes, the moves among the structure classes described as a Markov process, and the whole alignment process is described with a two-level Hidden Markov Model (HMM) outputting pairs of aligned characters. The model of a sequence pair is extended to progressive multiple alignment using a modification of the phylogeny-aware algorithm that distinguishes insertions from deletions (Löytynoja & Goldman 2005), a method that can be seen as a greedy ‘short cut’ towards full evolutionary alignment (e.g. Thorne *et al*. 1991; Hein *et al*. 2003; Holmes 2003).

We have implemented the method described in the alignment program prank. Analyses of real data show that the algorithm successfully uses different model states and the posterior probabilities for alternative structure classes in different parts of the alignment well match the known genomic structures of the sequences.

## 2. Material and methods

We have implemented our pairwise alignment algorithm for sequences with a structure as an extension of the homogeneous model that distinguishes insertions and deletions and handles insertions in an evolutionarily meaningful way (Löytynoja & Goldman 2005). Similar to the basic homogeneous model, the structure model can be extended to multiple alignments by iteration of pairwise alignment according to a guide tree, though, for clarity, we ignore the correction for pre-existing insertions here and present the algorithm for a standard pairwise alignment with affine gap penalties. General descriptions of HMMs and pair HMMs for sequence alignment are given by Rabiner (1989) and Durbin *et al*. (1998), respectively.

### (a) Model states and state transitions

The model can be seen as a two-level HMM (figure 1): on the higher level, the HMM consists of start and end states (S and E, respectively) and of two or more structure classes; on the lower level, each structure class h consists of three character-emitting states *X*_*h*_, *Y*_*h*_ and *M*_*h*_ emitting a character against a gap, a gap against a character and two characters matching, respectively, and a silent linker state (*W*_*h*_) connecting the two levels. Structure classes describe distinct evolutionary processes (such as a fast or slowly evolving site or region, or a codon site), and the moves between the classes define a sequence structure (i.e. regions/sites evolving differently); the moves within a structure class describe the character matching process within a given evolutionary process.

Probabilities *β*_*gh*_ for transitions between structure classes *g* and *h* (figure 1) are predefined and fixed. Transition within a structure is structure class specific: for structure class *h*, *δ*_*h*_ is the probability of moving to one of the gap states, and 1−2*δ*_*h*_ to a match state; *ϵ*_*h*_ and *γ*_*h*_ of staying in a gap state or in a match state, respectively; and 1−*ϵ*_*h*_ and 1−*γ*_*h*_ of moving back to the wait state *W*_*h*_. *ϵ*_*h*_ and *γ*_*h*_ are fixed (*γ*_*h*_=0 makes sites independent), whereas *δ*_*h*_ is jointly defined by a structure-specific insertion–deletion rate *r*_*h*_ and the evolutionary time

(2.1) $$\delta_{h}=1-{\text{e}}^{-r_{h}(|x|+|y|)},$$

where |***x***| and |***y***| are the evolutionary distances from the ancestral node to the two child nodes (either extant or reconstructed ancestral sequences) to be aligned (figure 2*a*).

### (b) Character emission and evolutionary match scores

We consider a pairwise alignment of sequences *x* and *y* consisting of characters *x*_1_ … *x*_*n*_ and *y*_1_ … *y*_*m*_. Sequence sites are described with vectors of probabilities, $p_{a}^{h}(x_{i})$, that the site *i* in sequence *x* has character *a* given that the process is in structure class *h*. If no sequence structure is imposed, the observed character at a terminal node is given a probability of 1 and others are set to 0; if the observed character is ambiguous, the probability is shared among different characters. For sequences with a known structure (e.g. gene annotation), character probabilities for some structure classes can be set positive and for other classes zero. At internal nodes, $p_{a}^{h}(x_{i})$ is defined from the pairwise alignment of the two child nodes as a conditional probability of all possible parent characters, given the child sites and all their descendants related by a phylogenetic tree and the process defined for structure class *h*.

Character emission is defined by an evolutionary substitution model (such as that of Jukes & Cantor (1969) or Hasegawa *et al*. (1985)) and the evolutionary distance between the parent and the child sequences. In state *M*_*h*_, a conditional probability for a parent character *a* at ancestral position *z*_*k*_, given the child character distributions, is defined by

(2.2) $$L_{z_{k}}^{\left(M_{h}\right)}(a)=\sum_{b}s_{ab}^{h}(x)p_{b}^{h}(x_{i})\sum_{c}s_{ac}^{h}(y)p_{c}^{h}(y_{j}),$$

where $s_{ab}^{h}(x)$ is the substitution probability between characters *a* and *b* given |***x***|, the evolutionary distance between sequence *x* and its immediate ancestor, and an evolutionary substitution model in structure class *h* (and similarly for $s_{ac}^{h}(y)$). As *z*_*k*_ cannot be known, the probabilities are summed over all possible character assignments *a* at the parent site, and an evolutionary score, $d_{x_{i},y_{j}}^{h}$, for a match in structure class *h* is obtained by dividing the probability of observed character emissions by the probability of the process emitting the same output randomly

(2.3) $$d_{x_{i},y_{j}}^{h}=\frac{\sum_{a}q_{a}^{h}L_{z_{k}}^{\left(M_{h}\right)}(a)}{\sum_{b}q_{b}^{N}p_{b}^{h}(x_{i})\sum_{c}q_{c}^{N}p_{c}^{h}(y_{j})},$$

where $q_{a}^{h}$ denotes the equilibrium frequency of character *a* in structure class *h*, and the superscript *N* denotes the homogeneous null model (i.e. no structure).

In states *X*_*h*_ and *Y*_*h*_, the probability depends only on the existing child and is defined by the equilibrium frequencies of the possible characters at the child site and their conditional probabilities, given the subtree below that child. The score for a gap in sequence *y*, $d_{x_{i},-}^{h}$, is given by

(2.4) $$d_{x_{i},-}^{h}=\frac{\sum_{a}q_{a}^{h}p_{a}^{h}(x_{i})}{\sum_{a}q_{a}^{N}p_{a}^{h}(x_{i})},$$

and is similarly defined for a gap in sequence *x*.

The expected number of insertions and deletions observed between two sequences depends on their evolutionary distance (equation (2.1)), whereas their length distribution is not expected to be time dependent. Typically, $d_{x_{i},-}^{h}$ and $d_{-,y_{j}}^{h}$ are close to 1, and the alignment is dominated by $d_{x_{i},y_{j}}^{h}$ and the expected similarity between the sequences given their evolutionary divergence.

The evolutionary modelling of substitution and insertion–deletion processes ensures that the structure classes are correctly scaled for the alignment of sequences that are differently diverged. The character substitution processes are described by instantaneous rate matrices (figure 2*b*), and given the evolutionary distances between the two nodes to align, substitution probability matrices that correctly reflect the expected divergence between the sequences are computed (figure 2*c*). Closely related sequences are expected to be similar just by their recent ancestry and, if the base frequencies in the structure classes are not drastically different, the sequences contain little information to distinguish the regions evolving under different processes.

However, correct homology between sequences, especially on nucleotide level that has low information content, may not be detectable even between moderately diverged sequence pairs (Pollard *et al*. 2004). By modelling the substitution process between the sequences aligned, our approach allows for setting an upper limit for the accepted pairwise distance and thus adjusting the sensitivity and specificity of the alignment. By setting the distance low, the sequence matching becomes stringent and, while it aligns the conserved parts normally, the method leaves the more uncertain diverged sequence regions unmatched.

### (c) Pairwise and progressive multiple alignment

Given the probabilities for state transitions and character emissions, two sequences are aligned by searching the most probable state path through the HMM. The algorithm finding the path is generally called Viterbi algorithm, and for a pair HMM similar to ours, it resembles the affine gap-cost algorithm of Gotoh (1982) as described by Durbin *et al*. (1998). Our approach differs from the standard algorithm in two respects: (i) the recursions are not only computed over the sequence sites *x*_0_ … *x*_*n*_ and *y*_0_ … *y*_*m*_, but also over the structure classes 1 … *r* such that the state transition probability *β*_*gh*_ contributes to the probability of each move, and (ii) we correctly allow for the moves between states *X*_*h*_ and *Y*_*h*_ indicating independent insertion–deletion events at the same or neighbouring sites.

The recursion for pairwise alignment with an affine gap penalty is described in algorithm A.1 (appendix), and its extension to progressive multiple alignment is straightforward (Löytynoja & Goldman 2005). For the latter, we define the probability vector $p_{a}^{h}(z_{k})$ for parent site *z*_*k*_ as the conditional probabilities of characters *a*, given the child sites in the pairwise alignment. Given equation (2.2) and defining $L_{z_{k}}^{\left(X_{h}\right)}(a)$ for the single child *x*_*i*_ as

(2.5) $$L_{z_{k}}^{\left(X_{h}\right)}(a)=\sum_{b}s_{ab}^{h}(x)p_{b}^{h}(x_{i}),$$

(and similarly $L_{z_{k}}^{\left(Y_{h}\right)}$ for the single child *y*_*j*_), in an internal node $p_{a}^{h}(z_{k})=L_{z_{k}}^{\left(\cdot \right)}(a)$, where · denotes *M*_*h*_, *X*_*h*_ or *Y*_*h*_ depending on which is the most probable character-matching event.

Given all the sites on the alignment path, the ancestral sequence is fully defined and can be aligned with another sequence. Ancestral sequences are technically not treated differently from extant ones.

### (d) Structure class posterior probabilities

The posterior probability of the process being in a certain state at a given moment is traditionally computed using forward/backward algorithms (Rabiner 1989). We use a similar approach to compute the posterior probabilities for alternative structure classes across the sites of a pairwise alignment.

For sites 1 … *l*, the probability of observing site *z*_*k*_, an ancestor for column *k* in the alignment, that either matches two sites or creates a gap using structure class *h* is given by

(2.6) $$L^{\left(\cdot \right)}(z_{k})=\sum_{a}q_{a}^{h}L_{z_{k}}^{\left(\cdot \right)}(a),$$

where · denotes *M*_*h*_, *X*_*h*_ or *Y*_*h*_ depending on which is the most probable character-matching event and $L_{z_{k}}^{\left(\cdot \right)}(a)$ is given by equations (2.2) and (2.5). Then, forward moves from the site *z*_*k*−1_ to a matching site *z*_*k*_, and to a site *z*_*k*_ that aligns *x*_*i*_ against a gap, in structure class *h* are defined as

(2.7) $$f^{h}(z_{k})=\sum_{g}f^{g}(z_{k-1})h_{\cdot M}^{gh}L_{h}^{\left(M_{h}\right)}(z_{k})\;\text{and}\;f^{h}(z_{k})=\sum_{g}f^{g}(z_{k-1})h_{\cdot X}^{gh}L_{h}^{\left(X_{h}\right)}(z_{k}),$$

respectively, where · denotes either *X*, *Y* or *M* depending if the previous site was one of the two gaps or a match, respectively (algorithm A.1). This is similarly defined for moves aligning the site *y*_*j*_ against a gap.

For the forward computation, the initialization and termination conditions are defined as in algorithm A.1 except that we denote them *f*^*h*^(*z*_0_) and *f*^*h*^(*z*_*l*+1_), respectively. For the backward computation, the initialization and termination terms, *b*^*h*^(*z*_*l*+1_) and *b*^*h*^(*z*_0_), simply change their places and then a backward move from a matching site *z*_*k*+1_ to the site *z*_*k*_ in structure class *h* is defined as

(2.8) $$b^{h}(z_{k})=\sum_{g}h_{\cdot M}^{hg}L_{g}^{\left(M_{g}\right)}(z_{k+1})b^{g}(z_{k+1}),$$

and is similarly computed for moves from sites aligning a site against a gap.

Given the forward and backward algorithms, the relative probability of being in structure classes *h* at the site *z*_*k*_ is

(2.9) $$P^{h}(z_{k})=\frac{f^{h}(z_{k})b^{h}(z_{k})}{f^{E}},$$

where *f*^*E*^ denotes the full probability of the forward recursion, i.e. the sum of all possible paths through the structure classes.

### (e) Alignment reliability

Our approach requires normalization of the match and gap scores (equations (2.3) and (2.4)) and does not allow for the computation of an unnormalized probability for a specific solution. However, we can still use forward and backward computation similar to Durbin *et al*. (1998), sum the probabilities of all possible moves in the alignment recursion (*cf. max*( ) in algorithm A.1) and calculate the proportion of the total score supporting the transition in the alignment path.

As the support score is defined for a given alignment solution, we sum the probabilities of transitions that give the same alignment of characters (i.e. moves to either *X*_*h*_, *Y*_*h*_ or *M*_*h*_) across all structure classes *h*. The score is computed for each transition on the Viterbi path in each pairwise alignment and, if the insertion-aware algorithm (Löytynoja & Goldman 2005) is used, the computation in ancestral nodes skips over the pre-existing insertions. The support score can be seen as a measure of the local reliability of a specific alignment solution.

## 3. Application

We have implemented the recursions described above in the alignment program prank that is downloadable under http://www.ebi.ac.uk/goldman/prank. Our implementation allows for defining different alignment HMMs in text files, such that the method can easily be adapted to the alignment of sequences from any alphabet using models of any complexity. Here, we describe results from alignment of genomic sequences.

### (a) Test data and alignment model

We aligned the CAPZA2 gene from 15 mammalian species using a simple nucleotide model and a more complex codon model. Genomic sequences for the protein coding region and 500 bases of upstream and downstream flanking sequence were extracted from the multiple alignment of encode target region 1 (The ENCODE Project Consortium 2007), and alignment gaps were removed. The alignment guide trees were based on the encode neutral tree.

A model is described by state transition probabilities *β*_*gh*_ and parameters $q_{a}^{h}$, $s_{ab}^{h}(x)$, *δ*_*h*_ and *ϵ*_*h*_ for the evolutionary processes in different structure classes. The alignment model fast/slow consists of two classes, *F* and *S*, describing fast and slowly evolving sequence sites. The average lengths of fast and slow regions are 200 and 50 bases, respectively, and the gap opening rate and the expected gap length are higher in the former (1/20 subst. versus 1/40 subst.; and 10 bases versus 2 bases, respectively). The transition–transversion ratio is set to 2 and character frequencies $q_{a}^{h}$ are defined by the empirical estimate *π* in both classes, whereas the instantaneous rate matrices (to define the substitution rate matrices $s_{ab}^{h}(x)$) are based on ***Q***: in class *S*, ***Q*** is scaled down giving a substitution rate that is 0.75 of the estimated rate, and in class *F,* it is scaled up such that, given the equilibrium distribution of the structure classes, the average rate of the model equals the estimated rate.

The alignment model codon is an extension of the fast–slow model and consists of five structure classes, the two single-character classes *S* and *F* and three consecutive nucleotide classes modelling a codon. The character-matching states in the three codon classes are connected and, when two characters are matched in the first class, characters have also to be matched in the second and third class; similarly, the lengths of alignment gaps are always multiples of three and gaps are only possible in phase 0. The average lengths of non-coding and coding sequences are 500 and 100 bases, respectively, and moves to and from a codon are only possible through the *S* state. The gap-opening rate and the expected gap length in the codon are 1/40 substitutions and 3 bases, respectively. The evolutionary process in states *S* and *F* is as described above; for the three codon sites, $q_{a}^{h}$ and the instantaneous rate matrices are defined by first computing the parameters for a codon with the selection parameter *ω* value 0.25 (Nielsen & Yang 1998), and then collapsing the parameters *π* and ***Q*** for the three distinct sites.

In both cases, the $q_{a}^{N}$ equals the empirical *π*.

### (b) Results

The true alignment for the given genomic region is obviously not known. Instead, we assume that the use of a structure state that more accurately describes the underlying evolutionary process produces improved alignments, and compare the posterior probabilities of being in different structure classes across the sequence sites to the known biological features, namely the 10 protein-coding exons.

The simplistic model fast/slow is able to identify the protein-coding exons along the alignment of human and mouse CAPZA2 sequences (figure 3*a*). However, the posterior probability curve is rather smooth and the accuracy of exon prediction based on any cut-off value would be poor. Also, the model describes single unlinked sites and many conserved non-protein-coding regions obtain high probabilities of being aligned by the slowly evolving class (such as 5′ and 3′ UTRs and sequence immediately flanking the exons). With the model codon, which adds three classes describing the periodicity of codon, the separation between the protein-coding exons and the conserved splicing signal becomes clearer, though parts of the 5′ and 3′ UTRs still obtain high probabilities for the codon classes (figure 3*b*). The model detects protein-coding regions purely based on the periodicity of substitution rates and gap lengths of multiples of three, and it may be misled by few random substitutions or gaps that happen to be in the right frame.

The performance of our method in the pairwise alignment of human and mouse seems satisfactory but the benefits of structure modelling should be more significant in multiple alignments. First, the alignments of closely related more similar sequences should provide information of the spatial variation of evolutionary processes and help the more difficult alignment of distantly related sequences. Second, multiple sequences provide more information of the sequence structure than two sequences only, and multiple closely related sequences can provide information on features that do not exist in a more distantly related sequence. As the method is progressive, information is generated for each internal node and can be used to study e.g. lineage-specific differences.

As expected, the alignment of very close sequences, such as human and chimpanzee, does not provide information on the sequence structure and, with the exception of long gaps, the posterior probabilities of different structure classes roughly reflect their background frequencies (not shown). On the other hand, the posterior probabilities for the codon classes in the alignment of five primate species rather accurately match the known protein-coding exons and provide an exon annotation comparable with that of the human–mouse pairwise alignment—with the difference that the former would potentially identify novel exons only existing in primates (figure 3*c* (i)).

The addition of the rest of the eutherian mammals (figure 3*c* (ii)) further sharpens the posterior probability curve at the exon boundaries but does not fully resolve the over-prediction of coding sequence in the beginning and end of the gene. Interestingly, the exon seven is consistently predicted to start 50 bases earlier than the true splice site (figure 3*c*). The upstream region is nearly identical all the way until monodelphis but a one-base insertion in mouse and rat suggests non-protein-coding function (not shown). The inclusion of monodelphis sequence would have only a marginal effect on the exon annotation, and the platypus sequence is incomplete and lacks the first exon.

Using alignment anchoring, the pairwise alignments of human and mouse sequences took approximately 600 and 1500 s on an AMD Opteron workstation when using the models fast/slow and codon, respectively. In multiple alignment, long insertion–deletions and missing data slow down the alignment significantly.

## 4. Discussion

We have developed an alignment method that allows for incorporating sequence structure information into the alignment process while still taking into account the evolutionary relatedness among the sequences. In contrast to an earlier approach extending the profile alignment (Edgar & Sjölander 2003), we base our method on progressive alignment and model the structural regions/sites with distinct evolutionary processes. Although our approach is not based on a full evolutionary model such as that of Arribas-Gil *et al*. (2007) and Satija *et al*. (2008), it is computationally less demanding and can be easily extended to describe large numbers of processes and biologically realistic sequence structures. The computational complexity of our approach naturally grows with the number of processes described, but our preliminary analyses have shown that in many cases even a moderate number of structure classes is able to capture a significant proportion of the evolutionary signal, such as nucleotide sequences' codon structure and more variable third positions. Also, the complexity reduces significantly when the structure classes are only sparsely connected, and we have successfully tested models with few tens of different classes.

## Figures and Tables

**Figure 1:**
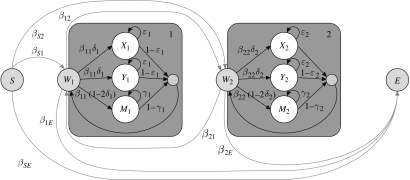
The simplest non-homogeneous alignment model consists of non-emitting start and end states (light grey circles; S and E) and two structure classes (grey boxes; 1 and 2), each describing an evolutionary process of its own. Moves between structure classes and moves within a structure class are denoted with grey and black arrows, respectively. For clarity, the moves from character emitting states (white circles; *X*_*i*_, *Y*_*i*_ and *M*_*i*_) back to a non-emitting linker state (light grey; *W*_*i*_) are drawn via a dummy state (light grey, empty circles).

**Figure 2:**
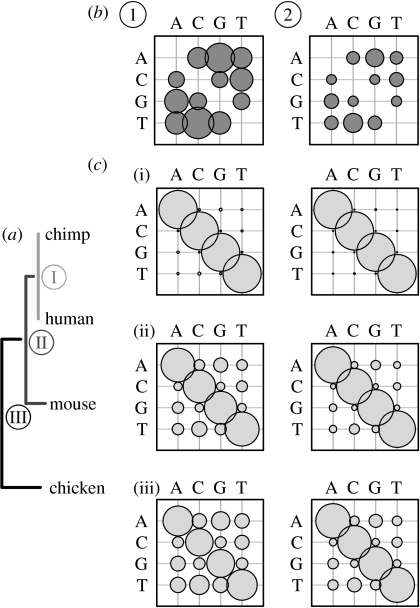
(*a*) A multiple alignment is built from pairwise alignments performed in order of decreasing relatedness (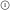, 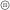 and 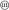), each alignment describing the ancestral node for the two nodes (extant or ancestral sequences) to be aligned. (*b*) The substitution process in each structure class is described by an instantaneous rate matrix ***Q***_*i*_, here indicated by plots 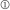 and 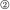 showing the rates between different nucleotides as relative sizes of bubbles. In this example, structure classes 1 and 2 model regions of DNA sequence that evolve at the rate that is 150 and 50 per cent of the average rate, respectively. (*c*) For each pairwise alignment, indicated by different shades in the tree (*a*), substitution probability matrices for every structure class are computed from the corresponding matrix ***Q***_*i*_. The evolutionary divergence between the sequence/ancestral node pairs to be aligned varies, as shown by the relative length of horizontal bars in the tree, and the alignments contain unequal amounts of information to distinguish the two evolutionary processes. (i) Between human and chimpanzee, both fast and slowly evolving regions (left and right matrix, respectively) are mostly conserved and the diagonal bubbles indicating no change are dominant. In the alignment of (ii) primate ancestor to mouse and (iii) mammalian ancestor to chicken, the fast evolving regions (left matrix) contain greater numbers of substitutions and the off-diagonal bubbles are relatively bigger.

**Figure 3:**
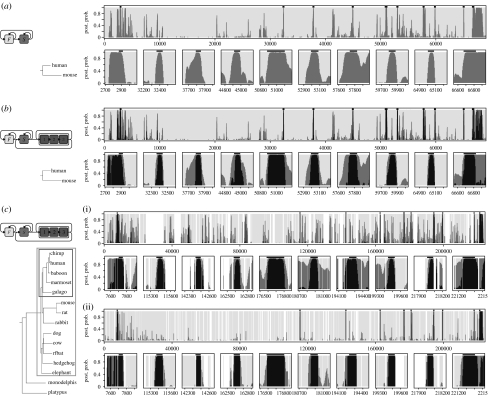
The panels in (*a*)–(*c*) show the posterior probability of different structure classes (top) across the full alignment and (bottom) around the known protein-coding exons. In (*a*) and (*b*), the models fast/slow and codon are used to align the human and mouse sequences; in (*c*), the model codon to align fifteen mammalian sequences. Light grey, dark grey and black represent the structure states modelling fast and slowly evolving sites and protein-coding regions, respectively. In (*c*), the addition of more distantly related sequences (dark grey and light grey frames in the tree correspond to panels in (i) and (ii) respectively) increases the evolutionary information and the high posterior probability for the codon states (in black) more accurately matches the locations of known exons. The known locations of the coding exons are marked with black bars (top). The empty gaps in the plots indicate insertions in some other part of the tree.

## Appendix A

An algorithm for pairwise alignment of sequences with structure. Initialization:

$$v_{h}^{\cdot }(i,-1),v_{h}^{\cdot }(-1,j)\;\text{are}\;\text{set}\;\text{to}\;0;\;v_{h}^{X}(0,0)=v_{h}^{Y}(0,0)=\beta_{Bh}\delta_{h};\;v_{h}^{M}(0,0)=\beta_{Bh}(1-2\delta_{h}).$$

Recursion:

i=0,…,n,j=0,…,m,except(0,0);g=1,…,r,h=1,…,r;

$$v_{h}^{X}(i,j)=d_{x_{i},-}^{h}\times \text{max}\{\begin{array}{cc} v_{g}^{X}(i-1,j)h_{XX}^{gh} & \;\text{if}\;(g=h)\\ v_{g}^{Y}(i-1,j)h_{YX}^{gh} & \;h_{XX}^{gh}=h_{YY}^{gh}=(\epsilon_{g}+(1-\epsilon_{g})\beta_{gh}\delta_{h})\\ v_{g}^{M}(i-1,j)h_{MX}^{gh} & \;h_{MM}^{gh}=\gamma_{g}+(1-\gamma_{g})\beta_{gh}(1-2\delta_{h}) \end{array}$$

$$v_{h}^{Y}(i,j)=d_{-,y_{j}}^{h}\times \text{max}\{\begin{array}{cc} v_{g}^{X}(i,j-1)h_{YX}^{gh} & \;\text{if}\;(g\neq h)\\ v_{g}^{Y}(i,j-1)h_{YY}^{gh} & \;h_{XX}^{gh}=h_{YY}^{gh}=(1-\epsilon_{g})\beta_{gh}\delta_{h}\\ v_{g}^{M}(i,j-1)h_{MY}^{gh} & \;h_{MM}^{gh}=(1-\gamma_{g})\beta_{gh}(1-2\delta_{h}) \end{array}$$

$$v_{h}^{M}(i,j)=d_{x_{i},y_{j}}^{h}\times \text{max}\{\begin{array}{cc} v_{g}^{X}(i-1,j-1)h_{XM}^{gh} & \begin{array}{l} \text{always }\\ h_{YX}^{gh}=h_{XY}^{gh}=(1-\epsilon_{g})\beta_{gh}\delta_{h} \end{array}\\ v_{g}^{Y}(i-1,j-1)h_{YM}^{gh} & \;h_{MX}^{gh}=h_{MY}^{gh}=(1-\gamma_{g})\beta_{gh}\delta_{h}\\ v_{g}^{M}(i-1,j-1)h_{MM}^{gh} & \;h_{XM}^{gh}=h_{YM}^{gh}=(1-\epsilon_{g})\beta_{gh}(1-2\delta_{h}) \end{array}$$

Termination:

$$v_{h}^{E}=\text{max}(v_{h}^{X}(n,m)(1-\epsilon_{h}),v_{h}^{Y}(n,m)(1-\epsilon_{h}),v_{h}^{M}(n,m)(1-\gamma_{h}))\beta_{hE}.$$
